# Persian Discectomy Successful Score (PDSS): a new evaluation measure for scoring successful surgery in lumbar disc herniation

**Published:** 2012-11

**Authors:** Parisa Azimi

**Affiliations:** ^a^Department of Neurosurgery, Shahid Beheshti University of Medical Sciences, Tehran, Iran.

## Abstract

**Background:**

Lumbar disc herniation (LDH) is a common cause of low back and radicular leg pains. This study aimed to evaluate the Persian Discectomy Successful Score (PDSS) in patients diagnosed with lumbar disc herniation undergoing discectomy. The PDSS first was developed based on the JOA score. It is a linear transformation of patients’ pre-operative JOA score that can predict the successful rate of surgery outcome in advance (PDSS= -1.036 × preoperative JOA + 23.635).

**Methods:**

A total of 103 patients with lumbar disc herniation submitted for discectomy operation were entered into the study during two years of study. A sensitivity analysis was carried out to examine the accuracy of the PDSS. For the analysis of the results, patients’ pre- and post-operative JOA score was used as gold standard to calculate the actual difference observed (JOA pre-operation minus JOA post-operation = DJOA) and to compare this measure (difference) with estimation derived from the PDSS.

**Results:**

The mean age of patients was 47.4 ± 9.7 (ranging from 17 to 79) years and were followed for at least three months. The difference between pre and postoperative JOA score (15.4 ± 3.7) was statistically significant (P less than 0.0001). The sensitivity, specificity and accuracy of the PDSS was 93.87%, 80%, and 93.2%, respectively. The PDSS had a positive predictive value of 98.9% and a negative predictive value of 40%.

**Conclusions:**

It seems that the preoperative JOA is able to predict discectomy results in patients with lumbar disc herniation. To confirm the findings of this study, a longitudinal study is recommended.

**Keywords:**

Discectomy Successful, lumbar disc herniation, Evaluation measure


					Volume 4, Suppl. 1 Nov 2012
Publisher: Kermanshah University of Medical Sciences
URL: http://www.jivresearch.org
Copyright:
Congress of Iranian Neurosurgeons, 14-16 November 2012, Kermanshah, Iran
Paper No. 24
Persian Discectomy Successful Score (PDSS): a new
evaluation measure for scoring successful surgery in lumbar
disc herniation
Parisa Azimi a, *
a
Department of Neurosurgery, Shahid Beheshti University of Medical Sciences, Tehran, Iran.
Abstract:
Background: Lumbar disc herniation (LDH) is a common cause of low back and radicular leg pains. This study aimed
to evaluate the Persian Discectomy Successful Score (PDSS) in patients diagnosed with lumbar disc herniation
undergoing discectomy. The PDSS first was developed based on the JOA score. It is a linear transformation of
patients' pre-operative JOA score that can predict the successful rate of surgery outcome in advance (PDSS= -1.036
x preoperative JOA + 23.635).
Methods: A total of 103 patients with lumbar disc herniation submitted for discectomy operation were entered into
the study during two years of study. A sensitivity analysis was carried out to examine the accuracy of the PDSS. For
the analysis of the results, patients' pre- and post-operative JOA score was used as gold standard to calculate the
actual difference observed (JOA pre-operation minus JOA post-operation = DJOA) and to compare this measure
(difference) with estimation derived from the PDSS.
Results: The mean age of patients was 47.4  9.7 (ranging from 17 to 79) years and were followed for at least
three months. The difference between pre and postoperative JOA score (15.4  3.7) was statistically significant (P
less than 0.0001). The sensitivity, specificity and accuracy of the PDSS was 93.87%, 80%, and 93.2%, respectively.
The PDSS had a positive predictive value of 98.9% and a negative predictive value of 40%.
Conclusions: It seems that the preoperative JOA is able to predict discectomy results in patients with lumbar disc
herniation. To confirm the findings of this study, a longitudinal study is recommended.
Keywords:
Discectomy Successful, lumbar disc herniation, Evaluation measure
* Corresponding Author at:
Parisa Azimi: Department of Neurosurgery, Imam-Hossain Hospital, Shahid-Beheshti University of Medical Sciences, Imam-Hossain sq., Tehran, Iran.
Tel: 021-77558081, Email: Parisa.azimi@gmail.com, (Azimi P.).

				

